# Problematic Internet use in Chinese adolescents and its relation to psychosomatic symptoms and life satisfaction

**DOI:** 10.1186/1471-2458-11-802

**Published:** 2011-10-14

**Authors:** Hui Cao, Ying Sun, Yuhui Wan, Jiahu Hao, Fangbiao Tao

**Affiliations:** 1 Anhui Provincial Key Laboratory of Population Health & Aristogenics, Anhui, China; 2 Department of Maternal, Child & Adolescent Health, School of Public Health, Anhui Medical University, NO. 81 Mei Shan Road, Hefei 230032, Anhui, China; 3 Department of General Medicine, School of Public Health, Anhui Medical University, 81 Mei Shan Road, Hefei 230032, Anhui, China

## Abstract

**Background:**

Problematic Internet use (PIU) is a growing problem in Chinese adolescents. Little is known about associations of PIU with physical and psychological health. This study was designed to investigate the prevalence of PIU and to test the relationships between PIU and psychosomatic symptoms and life satisfaction among adolescents in mainland China.

**Methods:**

A cross-sectional survey was conducted comprising a large representative sample of 17 599 students in eight cities of China. PIU was assessed by the 20-item Young Internet Addiction Test (YIAT). The Multidimensional Sub-health Questionnaire of Adolescents and the Multidimensional Students' Life Satisfaction Scale were administered to obtain information on psychosomatic symptoms and life satisfaction. Demographics and Internet usage patterns were also collected. Logistic regression was used to assess the effects of PIU on psychosomatic symptoms and life satisfaction.

**Results:**

Approximately 8.1% of subjects showed PIU. Adolescents with PIU were associated with males, high school students, urban, eastern and western areas, upper self-report family economy, service type mostly used for entertainment and relieving loneliness and more frequency of Internet use. Compared with normal Internet users, adolescents with PIU were more likely to suffer from psychosomatic symptoms (*P *< 0.001), including lack of physical energy (*P *< 0.001), physiological dysfunction (*P *< 0.001), weakened immunity (*P *< 0.001), emotional symptoms (*P *< 0.001), behavioural symptoms (*P *< 0.001) and social adaptation problems (*P *< 0.001). Adolescents with PIU had lower scores on total and all dimensions of life satisfaction (all *P *< 0.001). Adjusted for the demographic and Internet-related factors, there was positive significant relationship between PIU and psychosomatic symptoms, but negatively related to life satisfaction.

**Conclusions:**

PIU is common among Chinese students, and PIU was significantly associated with psychosomatic symptoms and life satisfaction. Effective measures are needed to prevent the spread of this problem and interventions to prevent the effects of PIU on psychosomatic symptoms and life satisfaction should be conducted as early as possible.

## Background

The Internet has become an important tool for social interaction, information, and entertainment [1]. However, as the Internet has moved into homes, schools, Internet cafes, and businesses, there has been a rapidly growing public awareness of the potential adverse effects arising from excessive, maladaptive or addictive Internet usage [2,3], which is a condition also known by terms such as problematic Internet use (PIU), Internet addiction, Internet dependence and pathological Internet use. Beard and Wolf defined PIU as use of the Internet that creates psychological, social, school, and/or work difficulties in a person's life [4].

As an important period between childhood and adulthood, adolescence is encompassed by alterations in physical, psychological, and social development [5]. During this developmental stage, more time is spent with peers and adults to face the variant social environment where more conflicts arise. The presence of relatively immature cognitive control [6,7], makes this period a time of vulnerability and adjustment and may lead to a higher incidence of affective disorders and addiction among adolescents [8,9]. Thus, adolescents are worthy of special consideration. International estimates of adolescent PIU vary widely. In Europe the prevalence has been reported to be between 1% and 9% [10,11], in the Middle East the prevalence is between 1% and 12% [12,13] and in Asia the prevalence has been reported to be between 2% and 18% [14-16]. According to the 27th China Internet development statistical report, as of 30 December 2010, there were 457 million people in China with access to the Internet. Of those, the group aged 10~29 years was the largest (57.1%) [17]. As one of the common mental health problems among Chinese adolescents, PIU is currently becoming more and more serious [18].

The negative impacts of PIU have progressively emerged. Recently, numerous studies had shown indulging in the use of the Internet is associated with a variety of problems. Kim et al [19] reported high-risk Internet users have inappropriate dietary behaviour and poor diet quality, which could result in stunted growth and development. Frangos et al [20] reported that PIU was also associated with other potential addictive personal habits of smoking, drinking alcohol or coffee, and taking drugs. Seo et al [21] found there were statistically significant positive correlations between Internet addiction and interpersonal problems (*r *= 0.43, *P *< 0.001). Ko et al [22] found that after controlling for the effects of shared associated factors, adolescents with Internet addiction were more likely to display aggressive behaviours. Kelleci et al [23] reported that Internet use in adolescents was associated with more severe psychiatric symptoms. Morrison et al [24] found that those who regard themselves as dependent on the Internet report high levels of depressive symptoms.

Although many studies on the associations between PIU and physical and psychological health have been carried out, some questions remain. First, some studies have recruited participants on-line or used a convenience sample [25,26]. These studies have inherent biases, which make it difficult to accurately assess the prevalence of PIU as well as the relationship between psychiatric symptoms and PIU. Second, many studies were often restricted and either referred to local or regional samples [27] which could not really be generalized, or examined only a part of the child and adolescent age spectrum [28]. Third, information on the effects of PIU on life satisfaction is limited.

For these reasons, we carried out a large-scale, cross-sectional study in China. The main purpose was to investigate the prevalence of PIU among adolescents aged 10 to 24, and to test the relationships between PIU and psychosomatic symptoms and life satisfaction. This study will contribute to our understanding of PIU among Chinese adolescents and help in designing educational policies to prevent PIU.

## Methods

### Procedure and sample

In March 2008, a school-based cross sectional survey was conducted in the high schools and college schools of eight cities in mainland China. According to geographic location and the level of economic development, the mainland China was classified three areas-- Eastern areas (high level of economic development), Middle areas (middle level of economic development) and Western areas (low level of economic development). In order to include the students with diversified socioeconomic backgrounds, the multi-stage stratified cluster sampling method was applied to obtain study subjects. Step 1, eight cities were samples from three areas. Based on a proportion of the population, three eastern cities, three middle cities and two western cities were selected. As follows: eastern (Beijing and Shaoxing in Zhejiang province, and Guangzhou in Guangdong province); middle (Ezhou in Hubei province, Harbin in Heilong-jiang province, and Taiyuan in Shanxi province), and western (Guiyang in Guizhou province and Chongqing) areas. Step 2, two junior high school (including one urban and one rural), two senior high school (including one urban and one rural) and one college in each city were randomly sampled as the target schools, and all the schools included were general junior and senior high schools and colleges (excluding experimental or key schools). Step 3, two classes from grades 7,8,10 and 11 in each high school (excluding grades 9 and 12 because of entrance examinations) and two classes from grades 1 and 2 in each college were randomly selected for the study. Step 4, all students in the selected classes were invited to participate in this research. A total of 17 622 students were recruited to participant in the study. Exclusion criteria of participants in this study included being older than 25 years of age, younger than 9 years of age and missing responses for Internet usage. Ultimately, 17 599 adolescents (9 015 males and 8 584 females), aged 10 to 24 years (mean age 16.1 years, SD = 2.8) were analyzed. Of these, 2 061did not use the Internet and the 15 538 who had Internet access provided usable information.

We used data from a large school-based cohort of the National High Technology Research and Development Program ('863' Program) of China. Written informed consent was obtained from the presidents of the schools, the teachers and the students. Ethical approval was obtained from Biomedicine Ethical Committee in Anhui Medical University.

### Instruments

A self-administered questionnaire containing information on socio-demographic indicators, Internet usage, psychosomatic symptoms and quality of life was filled in within a 20~30 min session in the classroom in the presence of the teachers, in order to minimize any potential information bias. The following socio-demographic characteristics were obtained: age, gender, grade, residential background (urban or rural areas), area and self-reported family economy. Internet usage pattern was assessed by examining the frequency of Internet use per week and the purpose of Internet use. The Young's 20-item Internet Addiction Test (YIAT) was applied in order to assess problematic Internet use. Each item is scored from 1 to 5, with 1 representing "not at all" and 5 representing "always". Hence, possible total scores range from 20 to 100. The following cut-off points were applied to the total YIAT score 1) Normal Internet use (NIU): scores 20~49; 2) Potential problematic Internet use (PIU): scores over 50 [29]. The split-half reliability and Cronbach alpha (α) coefficient was 0.86 and 0.90.

Psychosomatic symptoms were measured using the Multidimensional Sub-health Questionnaire of Adolescents (MSQA), which is a multidimensional, self-report symptom inventory developed by Tao et al [30] in China. MSQA consists of 71 items in total, which are divided into 6 symptom dimensions: lack of physical energy (11 items), physiological dysfunction (11 items), weakened immunity (10 items), emotional symptoms (17 items), behavioral symptoms (9 items) and social adaptation problems (13 items). Each item has six answer categories, in accordance with the duration of each symptom (none or last < 1 week, last ≥ 1 weeks, last ≥ 2 weeks, last ≥ 1 month, last ≥ 2 months, last ≥ 3 months). Because of the skewed nature of the distributions and the large proportions of adolescents reporting the duration of each symptom with none or last < 1 week on these items, each continuous symptom variable was transformed into a dichotomous variable. In the data analysis, no symptom and the symptom duration time < 1 month was assigned to 0, and the symptom duration time ≥1 month was assigned to 1. Then we calculated the score of total psychosomatic symptoms and six dimensions. The score of total and six dimensions were transformed into a dichotomous variable again, with the score ≥1 as positive dimension. It was one of the most widely used self-report screening tools for the purpose of investigating uncomfortable symptoms you actually feel during the last 3 months. The validity and reliability of the MSQA has been confirmed [31] and its test-retest reliability, Cronbach alpha (α) coefficient and split-half reliability coefficient were 0.87, 0.96 and 0.94, respectively.

Life satisfaction was assessed by the Multidimensional Students' Life Satisfaction Scale (MSLSS) which is a self-report symptom inventory developed by Huebner et al [32] and translated into Chinese [33]. It consists of 40 items in total, which are divided into 5 dimensions: family (7 items), friends (9 items), school (8 items), living environment (9 items) and self (7 items). Participants indicated their agreement with each of the statements on a 6-point scale ranging from disagree completely (1) to agree completely (6). Total scores and each dimension's score were calculated. Then, less than or equal to 25th percentile of the score was defined as dissatisfaction for each dimension. In logistic regression analyses, all dimensions of life satisfaction were used as the dichotomous variable. The validity and reliability of the MSLSS in China has been confirmed [33] and its test-retest reliability and Cronbach alpha (α) coefficients were 0.86 and 0.90.

### Data analysis

All the data were analyzed using SPSS version 13.0. Descriptive analyses were performed on all variables and the prevalence of PIU. Pearson chi-square test and independent-samples *t *test were used to compare the proportions and means of the independent variables versus dependent variables. Logistic regression analyses were performed to find out the effects of problematic Internet use (PIU) on psychosomatic symptoms and life satisfaction, controlling for gender, grade, residential background, area, self-report family economy, service type mostly used and frequency of Internet use. The independent (PIU) and dependent variables (psychosomatic symptoms and life satisfaction) were treated as a dummy variable coded 0 and 1 (0 for nonrisk and 1 for risk). The odds ratios (*OR*) and corresponding 95% confidence intervals (*CI*) were calculated. *P *< 0.05 was considered significant for all tests.

## Results

### General characteristics of subjects

The general characteristics of the participants and the relationships between PIU and general characteristics were provided in Table 1. Of the 15 538 students, there were 7 734 (49.8%) male and 7 804 (50.2%) female aged 10 to 24 years (mean age: 16.4 ± 2.8 years). If sorted by grades, there are 4 758 (30.6%) junior high school students (grades 7,8), 5 719 (36.8%) senior high school students (grades 10,11) and 5 061 (32.6%) college students (freshman and sophomore). More than half (59.5%) of the students came from urban. Mostly students (66.0%) reported middle family economy. Approximately 8.1% (1 262) were identified as problematic Internet users (PIUs) and males comprised 63.7% among them. More boys were PIUs than girls (10.4% vs 5.9%, *P *< 0.001). High school students were significantly more likely to be PIUs than college students (*P *< 0.001). Urban students reported more PIU than rural (*P *< 0.001). Adolescents reported upper family economy were more likely to be PIUs (10.1% vs 7.5%, *P *< 0.001) than adolescents reported normal family economy. The service type and frequency of Internet usage were also associated with PIU. The service type most often was entertainment (46.8%), followed by chatting (20.4%), information searching (14.7%), relieving loneliness (10.6%) and others (7.4%). Adolescents used Internet for relieving loneliness and entertainment were more likely to be PIUs and adolescents with more frequency of Internet use had a higher proportion of PIU (all *P *< 0.001).

**Table 1:** Sample characteristics of problematic Internet use (PIU), n (%)

Variables	Total	PIU	***χ***^***2***^	*P*
Gender				
Male	7 734(49.8)	804(10.4)	106.67	<0.001
Female	7 804(50.2)	458(5.9)		
Grade				
Junior High school	4 758(30.6)	395(8.3)	16.54	<0.001
Senior High school	5 719(36.8)	517(9.0)		
College	5 061(32.6)	350(6.9)		
Residential background				
Rural	6 297(40.5)	415(6.6)	33.28	<0.001
Urban	9 241(59.5)	847(9.2)		
Area				
Eastern area	5 866(37.8)	570(9.7)	84.56	<0.001
Middle area	5 254(33.8)	279(5.3)		
Western area	4 418(28.4)	413(9.3)		
Self-report family economy				
Lower	2 746(17.7)	237(8.6)	19.16	<0.001
Middle	10 250(66.0)	769(7.5)		
Upper	2 542(16.4)	256(10.1)		
Service type mostly used				
Information searching	2 291(14.7)	42(1.8)	361.52	<0.001
Entertainment	7 279(46.8)	764(10.5)		
Chatting	3 165(20.4)	145(4.6)		
Relieving loneliness	1 652(10.6)	254(15.4)		
Others	1 151(7.4)	57(5.0)		
Frequency of Internet use				
≤ 4 times/week	13 056(84.0)	770(5.9)	541.92	<0.001
≥ 5 times/week	2 482(16.0)	492(19.8)		

### PIU and psychosomatic symptoms, life satisfaction

Table 2 showed the prevalence of psychosomatic symptoms among adolescents with problematic Internet use and normal Internet use. The total psychosomatic symptoms and all 6 dimensions were significantly associated with PIU in *χ*^2 ^tests (all *P *< 0.001). Compared with normal Internet use, adolescents with PIU were more likely to suffer from psychosomatic symptoms, lack of physical energy, physiological dysfunction, weakened immunity, emotional symptoms, behavioural symptoms and social adaptation problems.

**Table 2:** The prevalence of psychosomatic symptoms among adolescents with problematic Internet use (PIU) and normal Internet use (NIU), n (%)

Symptom Dimension	NIU (n = 14 276)	PIU (n = 1 262)	***χ***^***2***^	*P*
Lack of physical energy	1 483 (10.4)	377 (29.9)	417.77	<0.001
Physiological dysfunction	2 325 (16.3)	459 (36.4)	318.04	<0.001
Weakened immunity	2 052 (14.4)	382 (30.3)	221.77	<0.001
Emotional symptoms	3 240 (22.7)	711 (56.3)	692.14	<0.001
Behavioral symptoms	1 837 (12.9)	526 (41.7)	746.44	<0.001
Social adaptation problems	4 004 (28.0)	816 (64.7)	726.36	<0.001
Psychosomatic symptoms	6 074 (42.5)	951 (75.4)	503.89	<0.001

The mean total MSLSS score and the scores of the 5 dimensions in PIUs were 163.90 (SD = 23.48), 31.45 (SD = 7.95), 38.29 (SD = 5.78), 29.21(SD = 7.40), 33.44 (SD = 7.02) and 31.51 (SD = 6.42), respectively. Not surprisingly, PIUs had significantly lower scores on total and all dimensions (all *P *< 0.001) (Table 3).

**Table 3:** The mean scores (SD) of life satisfaction among adolescents with problematic Internet use (PIU) and normal Internet use (NIU), (mean ± SD).

Life Satisfaction Dimension	Total Mean Score	NIU (n = 14 276)	PIU (n = 1 262)	*t*	*P*
Family	34.94 ± 6.62	35.25 ± 6.39	31.45 ± 7.95	19.77	<0.001
Friends	40.86 ± 5.50	41.08 ± 5.41	38.29 ± 5.78	17.49	<0.001
School	34.07 ± 6.99	34.50 ± 6.78	29.21 ± 7.40	26.35	<0.001
Living environment	36.69 ± 7.35	36.97 ± 7.30	33.44 ± 7.02	16.53	<0.001
Self	33.45 ± 5.51	33.62 ± 5.39	31.51 ± 6.42	13.13	<0.001
Total	180.00 ± 23.47	181.42 ± 22.93	163.90 ± 23.48	25.98	<0.001

When the demographic and Internet-related factors were controlled, all dimensions of psychosomatic symptoms and life satisfaction were significantly associated with PIU. Table 4 indicated that there was positive significant relationship between PIU and psychosomatic symptoms, but negatively related to life satisfaction.

**Table 4:** The association problematic Internet use and psychosomatic symptoms and life satisfaction

Variables	Crude *OR *(95%*CI*)	Adjusted *OR *(95%*CI*) *
Psychosomatic symptoms		
Lack of physical energy	3.68 (3.22 ~ 4.19) **	3.50 (3.03 ~ 4.03) **
Physiological dysfunction	2.94 (2.60 ~ 3.32) **	2.83 (2.49 ~ 3.22) **
Weakened immunity	2.59 (2.27 ~ 2.94) **	2.33 (2.04 ~ 2.67) **
Emotional symptoms	4.40 (3.91 ~ 4.95) **	4.11 (3.62 ~ 4.66) **
Behavioral symptoms	4.84 (4.28 ~ 5.47) **	4.55 (3.99 ~ 5.20) **
Social adaptation problems	4.69 (4.16 ~ 5.30) **	4.23 (3.72 ~ 4.81) **
Life satisfaction^#^		
Family	2.81 (2.50 ~ 3.16) **	2.38 (2.10 ~ 2.70) **
Friends	2.39 (2.13 ~ 2.69) **	2.26 (2.00 ~ 2.55) **
School	3.79 (3.37 ~ 4.26) **	3.16 (2.79 ~ 3.57) **
Living environment	2.40 (2.14 ~ 2.70) **	2.28 (2.02 ~ 2.58) **
Self	2.05 (1.82 ~ 2.30) **	1.90 (1.69 ~ 2.15) **

*Adjusted for gender, grade, residential background, area, self-report family economy, service type mostly used and frequency of Internet use.

^# ^Less than or equal to 25th percentile of the score was defined as dissatisfaction for each dimension of life satisfaction.

** *P *< 0.001

## Discussion

The information provided here may help us better understand the prevalence of PIU and the relationships between PIU and psychosomatic symptoms, life satisfaction among Chinese adolescents. This study showed that 8.1% of the total study population, including 8.3% of junior high school subjects, 9.0% of senior high school subjects and 6.9% of college subjects, had PIU, which were consistent with many previous studies[34,35]. Lam et al [34] reported that 10.8% were diagnosed as Internet addicted users among high school students, and Ni et al [35] identified 6.4% of 3 557 first-year university students as Internet addicted. During adolescence, high school students usually experience dramatic serious problems than do individuals of other ages if they engage in problem behaviours. Thus, there is increasing evidence that PIU among high school students is emerging due to easy access to the Internet [12,36], similar to the current study which shown PIU may be more severe among high school students in China. The rate of Internet addiction among adolescents varies widely, from 1.0% to 36.7% [10-16,37]. There could be many reasons for this. First, different social and cultural contexts, as well as differing definitions and criteria for measuring Internet addiction, make it difficult to effectively compare findings. Second, the cut-off criteria for most studies were set without clinically established diagnostic validity. However, the current study used the YIAT to establish good diagnostic validity for the cut-off point. Finally, prevalence rates have varied widely depending on the sample. The above-mentioned most studies were limited by relatively small or unrepresentative samples, whereas the present study examined a nationwide sample of adolescents and confirmed the prevalence of PIU within adolescents aged 10 to 24.

Previous studies identified gender as a risk factor for PIU [1,34], and our results are in agreement with them. One explanation for this may be that males are more likely to play online games, engage in cybersex, view cyberporn, and gamble online, all of which are associated with addictive Internet use [38]. This study showed that the frequency spent using the Internet per week was associated with PIU. One explanation for the association between frequency of Internet usage and PIU is that it may be as much a symptom as it is a cause.

The present results showed that adolescents with PIU had more severe psychosomatic symptoms than those without. This finding also corresponds to analogous investigations of excessive Internet use [39,40], which has been reported that extensive Internet use may bring forth a heightened level of psychological arousal, possibly resulting in online users experiencing health problems. Psychosomatic symptoms in adolescence might be important signals of mental health and this should be taken seriously in school health and in general primary care.

Teenagers tend to use the Internet as a medium for socialising, but PIU can result in individuals spending ever-increasing amounts of time in online activities, leading to social withdrawal, self-neglect, poor diet and family problems. The consequences of PIU are insidious, becoming apparent after months of PIU and eventually engulfing all aspects of the individual's life. Teenagers with PIU have reportedly become physically aggressive when parents try to remove them from the computer. Late night use of the Internet can cause sleep deprivation and fatigue, which can adversely affect academic performance and can result in reversed sleep pattern and poor academic performance [41].

In the long term, problematic Internet use can cause serious health problems. Repetitive strain injury and back ache are common complaints. There have been at least 10 reports from China of users collapsing and dying following several days of uninterrupted online video game playing [41]. A sedentary life-style can increase risk of deep vein thrombosis and pulmonary embolus, eventually leading to obesity and its associated complications [42,43]. Similar to the study results above, our study found adolescents with PIU were more likely to suffer from physical symptoms, such as lack of physical energy, physiological dysfunction and weakened immunity.

In addition, the current study found adolescents with PIU were more likely to suffer from emotional symptoms, behavioural symptoms and social adaptation problems, meanwhile adolescents with PIU had significantly lower scores on total and all dimensions of life satisfaction. Adjusted for the demographic and Internet-related factors, all dimensions of psychopathological symptoms and life satisfaction were significantly associated with PIU. Given the essential role of the Internet in communication, recent other studies also have examined the influence of Internet use on psychological well-being. Several cross-sectional questionnaire and interviewing studies claim the underlying psychopathology of Internet addiction, including attention deficit and hyperactivity disorder (ADHD), depression, social anxiety, and substance dependence [44-48], in adolescents and college students. There are several possible mechanisms explaining this association. A co-morbid mental disorder may result in, contribute to, or exacerbate the symptoms of PIU. PIU may lead to, contribute to, or exacerbate the symptoms of various mental disorders. There may be underlying biological, psychological, and sociological mechanisms shared by PIU and various mental disorders.

### Strengths and limitations

Some strengths and limitations should be noted. First of all, it was a cross-sectional design, which does not allow for causality or the direction of relationships to be determined. However, appropriate analysis of cross-sectional data represents a useful initial step in identifying associations between PIU and psychosomatic symptoms, life satisfaction. Second, all information was obtained from a self-reported questionnaire, resulting in the possibility of response bias. Multiple assessments, interviews, and informants may have provided a richer and more thorough understanding of PIU. Third, since the survey was administered during class time, it is possible that some students, especially those addicted to the Internet, were absent from class when the questionnaire was administered. Therefore, the survey may have under-represented PIU by failing to record the responses of those who are so consumed by the Internet that they rarely leave their rooms, thus leading to an underestimation of the prevalence of PIU. Fourth, although the Young Internet Addiction Test (YIAT) is widely used to measure the severity of PIU, there is no data regarding psychometrics of the Chinese version of YIAT. So the cut-off of the YIAT identified among European population was used in this study, this may not be the same as that in Chinese population.

Despite limitations, this study investigated the prevalence of PIU and examined the associations between PIU and psychosomatic symptoms and life satisfaction based on data from a nationally representative sample of adolescents in mainland China. This study suggested that PIU was fairly common among Chinese adolescents, especially among high school students, and that PIU was significantly associated with psychosomatic symptoms and life satisfaction. The health department of the China government has now recognised PIU as a serious public health problem. Arguably, it is time for the World Health Organization and health departments around the world to develop effective health policies to increase public awareness of PIU. If we fail to acknowledge PIU as a public health issue, then it will continue its silent, endemic spread, affecting millions of children and adults, and eventually affecting societies and economies.

## Conclusions

In the information age, Internet use is becoming increasingly significant in the acquisition of information and the sharing of knowledge. Internet use is now considered to form part of the culture of adolescents, and hence studying Internet use and its negative aspects is tremendously important to the creation of a sound youth culture. This requires studies on the relationships between PIU and physical and psychological health. This present study has demonstrated that PIU was common among Chinese students, and PIU was significantly associated with psychosomatic symptoms and life satisfaction. Effective measures are needed to prevent the spread of this problem and interventions to prevent the effects of PIU on psychosomatic symptoms and life satisfaction should be conducted as early as possible.

## Abbreviations

PIU: Problematic Internet use; NIU: Normal Internet use; PIUs: Problematic Internet users; YIAT: Young's Internet Addiction Test; MSQA: Multidimensional Sub-health Questionnaire of Adolescents; MSLSS: Multidimensional Students' Life Satisfaction Scale; ADHD: Attention deficit and hyperactivity disorder; *CI*: Confidence interval; *OR*: Odds ratio; *χ*^2^: Chi square.

## Competing interests

The authors declare that they have no competing interests.

## Authors' contributions

HC performed the statistical analyses, interpreted the findings and drafted the manuscript. YS participated in study design, subjects' recruitment and data collection. YHW directed subjects' recruitment and data collection and participated in manuscript preparation. JHH participated in the statistical analyses and result interpretation. FBT obtained funding, designed the study, oversaw the study project, and participated in result interpretation and manuscript preparation.

All authors read and approved the submitted manuscript.

## Pre-publication history

The pre-publication history for this paper can be accessed here:

http://www.biomedcentral.com/1471-2458/11/802/prepub
